# Ethnic Differences in Arterial Wave Reflection Are Mostly Explained by Differences in Body Height - Cross-Sectional Analysis of the HELIUS Study

**DOI:** 10.1371/journal.pone.0160243

**Published:** 2016-07-29

**Authors:** Daan W. Eeftinck Schattenkerk, Jacqueline van Gorp, Marieke B. Snijder, Aeilko H. Zwinderman, Charles O. Agyemang, Ron J. G. Peters, Bert-Jan H. van den Born

**Affiliations:** 1 Department of Vascular Medicine, Academic Medical Center, Amsterdam, the Netherlands; 2 Department of Public Health, Academic Medical Center, Amsterdam, the Netherlands; 3 Department of Clinical Epidemiology and Biostatistics, Academic Medical Center, Amsterdam, the Netherlands; 4 Department of Cardiology, Academic Medical Center, Amsterdam, the Netherlands; Shanghai Institute of Hypertension, CHINA

## Abstract

**Background:**

Differences in arterial wave reflection and central blood pressure (BP) have been associated with cardiovascular disease (CVD) in various populations and may contribute to ethnic differences in CVD. Whether ethnic differences in wave reflection and central BP can be explained by conventional risk factors for CVD or may result from physiological differences remains undetermined.

**Methods:**

We examined ethnic differences in augmentation index (AIx) and central systolic BP and their determinants in a large multi-ethnic cohort study in Amsterdam, the Netherlands. A total of 8812 (46% male) participants aged 18–70 years of Dutch, South-Asian Surinamese, African Surinamese and Ghanaian origin were included. AIx and central BP were measured in duplicate using the Arteriograph system.

**Results:**

AIx and central systolic BP were significantly higher in South-Asian Surinamese (35±17%, 126±22 mmHg), African Surinamese (33±17%, 129±23 mmHg) and Ghanaian (33±16%, 135±24 mmHg) as compared with Dutch (27±17%, 118±20 mmHg, all p<0.001). Correction for cardiovascular risk factors only slightly reduced the difference in AIx, whereas correction for body height attenuated age and gender corrected ethnic differences in AIx the most. Differences in central systolic BP were primarily determined by differences in AIx for South-Asian Surinamese and by differences in peripheral systolic BP for subjects of African origin.

**Conclusions:**

Substantial differences in AIx and central BP exist across different ethnic groups that cannot be explained by differences in conventional risk factors for CVD. These findings may explain part of the underestimation of cardiovascular risk observed in populations of African and South-Asian descent.

## Introduction

Ethnic disparities in the burden of cardiovascular disease (CVD) are well recognized, but incompletely understood.[1–3] These disparities may be related to differences in arterial wave reflection and central blood pressure (BP). Independent of BP indices of wave reflection have been associated with CVD in different populations.[4–6] In addition, mounting evidence suggests that central (i.e. aortic) BP is a stronger predictor for future CVD than brachial BP as measured in daily practice and may be affected by arterial wave reflection.[7–10] Previous studies have reported higher augmentation index (AIx) and central BP among different ethnic groups compared to subjects of Western-European descent.[11–14] However, the contribution of conventional risk factors and the physiological origin of differences in AIx and central BP remain undetermined. Hypertension, smoking and diabetes have been independently associated with ethnic differences in AIx.[11,15] However, physiological factors including body height,[16,17] heart rate,[18,19] large artery stiffness,[20] peripheral resistance,[21] and stroke volume [22] have been shown to affect arterial wave reflection and could contribute to ethnic differences in AIx and central BP.

In the present study, we investigated differences in AIx and central BP in a large multi-ethnic population in Amsterdam, the Netherlands. Our principal aim was to assess the contribution of conventional cardiovascular risk factors and physiological determinants on ethnic differences in AIx as a BP independent measure of wave reflection, and secondary to assess the influence of differences AIx on variations in central BP.

## Methods

### Study Population

We used baseline data from the HEalthy LIfe in an Urban Setting (HELIUS) study, a large multi-ethnic population study carried out by the Academic Medical Center, Amsterdam, and the Public Health Service on health and health-care utilization among the six major ethnic groups residing in Amsterdam, the Netherlands. Details of the HELIUS study have been previously described in detail.[23] The HELIUS study is conducted in accordance with the Declaration of Helsinki and has been approved by the Ethical Review Board of the Academic Medical Center, Amsterdam. All participants provided written informed consent. For the present analysis, data collected from January 2011 until December 2014 were used from participants of Dutch (n = 2838), South-Asian Surinamese (n = 2383), African Surinamese (n = 2938) and Ghanaian (2189) origin, totaling 10.348 individuals who underwent a physical examination and in whom questionnaire data were available. Within this group we only included participants with available data on AIx and central BP, which resulted in a dataset of 8812 participants aged 45.5±13.0 years (49% male) for the current analyses. This included 2431 Dutch, 1928 South-Asian Surinamese and 2501 African-Surinamese (both first and second generation), and 1952 participants of Ghanaian descent (exclusively first generation). Missing data were primarily due to logistic reasons or failure to obtain valid readings.

### Study Procedures

Information on demographics, smoking behaviour, alcohol intake, and history of diseases was obtained by questionnaire. Participants were asked to bring their prescribed medications to the research location, which were coded according to the Anatomical Therapeutic Chemical (ATC) classification. Diabetes was defined as a fasting glucose level ≥7.0 mmol/l, or the use of glucose lowering medication. Hypertension was defined as brachial BP ≥140 systolic or ≥90 mmHg diastolic while seated, or the use of blood pressure lowering medication. Height and weight were recorded and body mass index (BMI) was calculated as weight (kg) divided by height squared (m^2^).

### Augmentation Index, Central Blood Pressure and Pulse Wave Velocity

Study participants visited the research location in the morning after an overnight fast and were asked to refrain from smoking the morning prior to the visit. Measurements were performed after at least 10 minutes of rest in supine position. The Arteriograph system (Tensiomed Kft., Budapest, Hungary) was used to assess AIx, central BP, brachial BP and pulse wave velocity (PWV). The Arteriograph system is an operator-independent non-invasive device that applies an oscillometric, occlusive technique by use of an upper-arm cuff to register brachial pressure curves. Its’ methodology and validation is described in detail elsewhere.[24–26] Arteriograph AIx has close correlation with the widely applied Sphygmocor (AtCor Medical Pty Ltd, West Ryde, Australia) AIx (r = 0.89, p<0.001);[26] invasively measured AIx (r = 0.90, p<0.001), central BP (r = 0.95, p<0.001) and PWV (r = 0.91, p<0.001).[25] All Arteriograph measurements were performed in duplicate and the results were averaged for further analysis.

### Systemic Vascular Resistance, Stroke Volume and Heart Rate

Hemodynamics were assessed by volume-clamp photoplethysmography with the Nexfin™ device (Edwards Lifesciences BMEYE, Amsterdam, the Netherlands).[27] This device uses the Finapres method [28] to continuously and non-invasively record finger arterial BP and reconstructed brachial BP.[29,30] Recordings were made at 200Hz using a finger-cuff placed around the mid-phalanx of the third finger. Mean arterial pressure (MAP) was calculated from the true integral of the arterial pressure wave over one beat divided by the corresponding inter-beat interval. Heart rate was the inverse of the inter-beat interval. Stroke volume (SV) was determined by the pulse contour method (Nexfin CO-trek).[31] Cardiac output (CO) was SV divided by the inter-beat interval. Systemic vascular resistance (SVR) was the ratio of MAP and CO. All Nexfin hemodynamic parameters were calculated from the average of a 1 minute period of stable recording.

### Statistical Analyses

Estimates of AIx and central systolic BP according to age were depicted for the four ethnic groups by spline interpolation using four degrees of freedom. A general linear model was used to compare AIx and central systolic BP between ethnic groups, pairwise comparisons were made according to least-significant-difference (LSD) and differences were reported as mean±SE. To assess the potential effects of antihypertensive therapy on ethnic differences in AIx and central systolic BP, we additionally performed a sensitivity analysis including antihypertensive drug naïve subjects exclusively. To explore the contribution of peripheral BP and AIx to ethnic differences in central BP, differences in central BP across ethnic groups were corrected for differences in peripheral BP and AIx with Dutch acting as reference. AIx was selected as a BP independent determinant of ethnic differences in central BP to explore the contribution of different covariates using three models. Model 1 was used to correct for age and gender. In model 2 differences in AIx were additionally corrected for systolic BP, BMI, total cholesterol, smoking and diabetes to explore the contribution of conventional CVD risk factors. Model 3 was used to additionally correct for body height, PWV, heart rate, SV and SVR. The physiological factors from model 3 were also individually included next to age and gender, to assess their relative contribution to ethnic differences in AIx. Furthermore, linear regression analysis was performed to test the contribution of covariates from model 3 to variations in AIx within each ethnic subgroup. Because of potential multicollinearity in regression analyses we assessed variance inflation factors for the various models and ethnic subgroups, in all instances these were <5 which indicates an acceptable level of potential multicolinearity.[32] Finally, we compared AIx between ethnic groups in young (≤30 years) and elderly (≥60 years) subjects to further explore the contribution of ageing to differences in AIx. In these subgroups we assessed the effects of physiological factors (model 3) on ethnic differences in AIx.

A p-value <0.05 was considered statistically significant. Statistical analyses were performed using SPSS (Version 20.0, IBM corp., Armonk, NY, USA). R (*V*ersion 2.15, *R Foundation for Statistical Computing*, *Vienna*, *Austria*) and GraphPad Prism (Version 5.00, GraphPad Software, San Diego, California, USA) were used for graphical representation of data.

## Results

**Fig 1:**
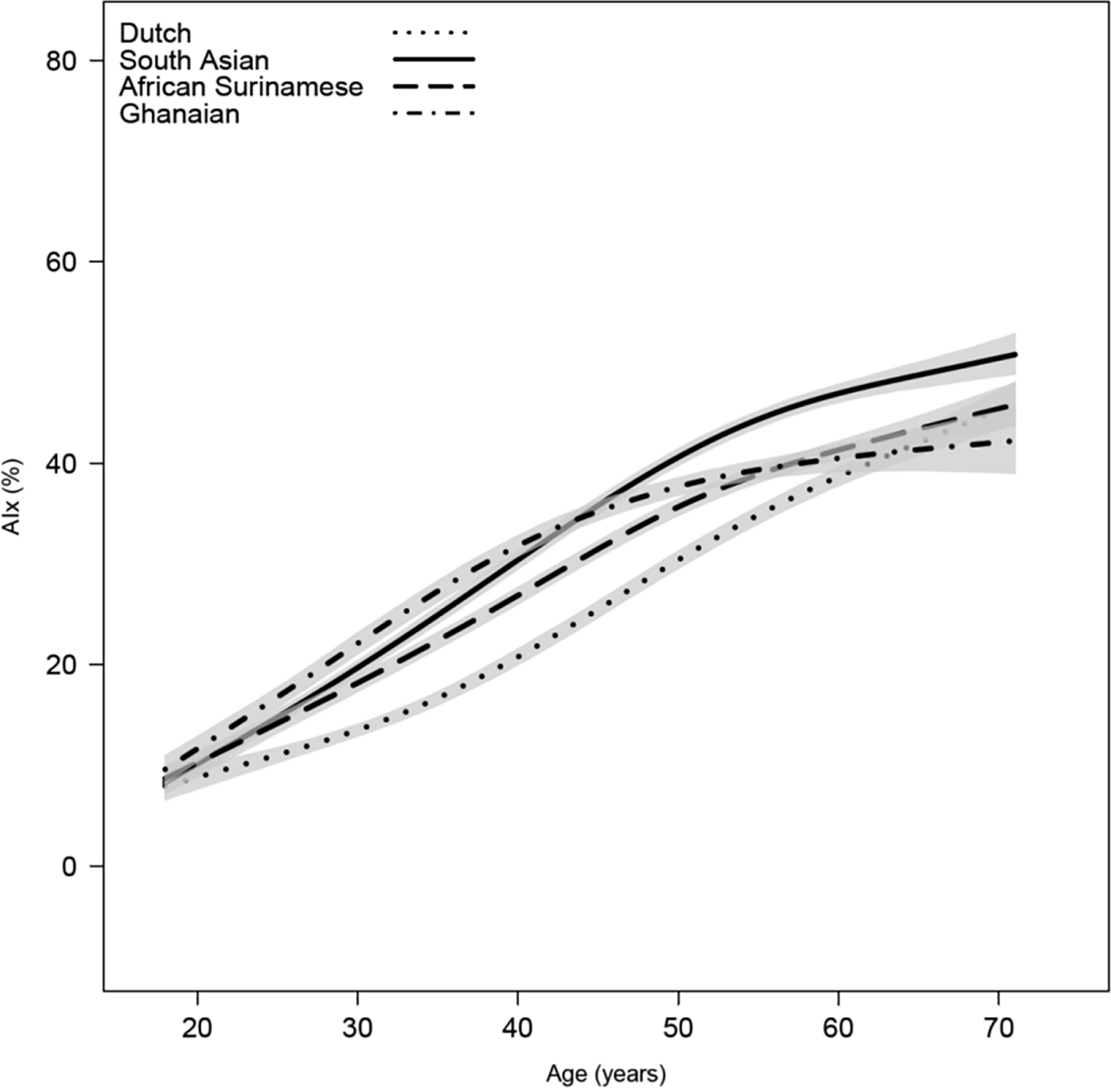
Augmentation index (AIx) for the different ethnic groups according to age. Gray shaded area denotes 95% CI of the regression line.

**Fig 2:**
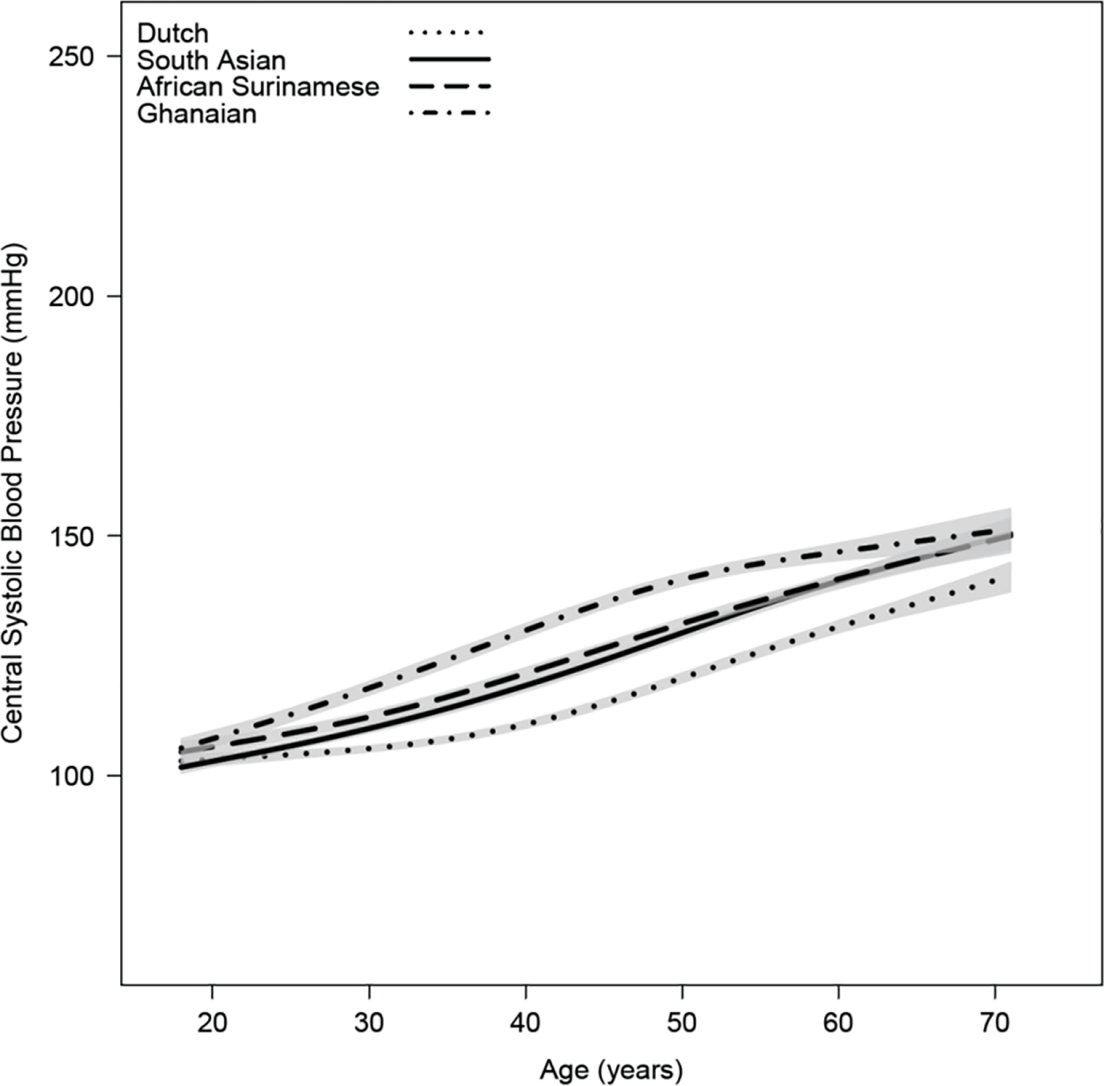
Central BP for the different ethnic groups according to age. Gray shaded area denotes 95% CI of the regression line.

General characteristics and hemodynamics of the study participants stratified by ethnicity are shown in Table 1. AIx and central BP for the different ethnic groups are shown in Figs 1 and 2, respectively. Both AIx and central systolic BP were markedly higher in South-Asian Surinamese (35±17%, 126±22 mmHg), African Surinamese (33±17%, 129±23 mmHg) and Ghanaian (33±16%, 135±24 mmHg) as compared with Dutch (27±17%, 118±20 mmHg, all pairwise p<0.001). In antihypertensive drug naïve subjects (n = 6952) AIx and central BP were lower in all groups, yet ethnic differences remained comparable: South-Asian Surinamese (32±17%, 121±20 mmHg), African Surinamese (30±16%, 125±21 mmHg) and Ghanaian (30±16%, 129±23 mmHg) versus Dutch (25±16%, 116±18 mmHg, all pairwise p<0.001). Figs 1 and 2 show the crude estimates of AIx and central systolic BP according to age for the four ethnic groups using spline interpolation. The age-related increase in AIx was most pronounced for South-Asian Surinamese and Ghanaians followed by African Surinamese. The difference in AIx with African Surinamese and Ghanaians became smaller after the age of 50, decreasing to values comparable with Dutch. Likewise, central systolic BP was higher in all ethnic minority groups compared to Dutch, showing similar curves in South-Asian and African Surinamese. Differences in central systolic BP as compared to Dutch were primarily explained by differences in AIx for South-Asian Surinamese and by differences in peripheral systolic BP for subjects of African descent. Correction for AIx resulted in a central systolic BP difference of 0.1±0.5 mmHg for South-Asian Surinamese (p = 0.93), 5.6±0.5 mmHg for African Surinamese (p<0.001) and 10.2±0.5 mmHg for Ghanaians (p<0.001) compared to Dutch. While with correction for peripheral systolic BP differences in central systolic BP were 3.1±0.2 mmHg for South-Asian Surinamese, 1.4±0.2 mmHg for African Surinamese and 1.1±0.2 mmHg for Ghanaians compared to Dutch (all p<0.001).

**Table 1:** Characteristics of the study population stratified by ethnicity.

	Dutch (n = 2431)	South-Asian Surinamese (n = 1928)	African Surinamese (n = 2501)	Ghanaians (n = 1952)	p-value
**Age,** y	46 ± 14	46 ± 13	48 ± 13	46 ± 11	
**Men,** %	51	53	41	44	
**Height**, cm	176 ± 9	166 ± 9	169 ± 9	166 ± 8	
**BMI,** kg/m²	25 ± 4	26 ± 4	27 ± 5	28 ± 5	
**Hypertension, %**	25	40	45	55	
**Smoking,** %	26	28	32	5	
**Diabetes,** %	3	19	11	13	
**HbA1c,** mmol/mol	36 ± 5	43 ± 10	40 ± 11	40 ± 10	All p<0.001
**AIx,** %	26 ± 17	34 ± 17	32 ± 17	33 ± 16	
**SBPao,** mmHg	118 ± 20	126 ± 22	132 ± 18	134 ± 24	
**SBPbr,** mmHg	125 ± 16	130 ± 18	139 ± 22	136 ± 19	
**DBPbr,** mmHg	77 ± 10	80 ± 10	82 ± 11	85 ± 11	
**HR,** beats/min	60 ± 9	63 ± 9	63 ± 10	64 ± 10	
**SV,** ml	93 ± 21	89 ± 20	88 ± 22	88 ± 17	
**SVR,** dynes/sec/cm^5^	1484 ± 585	1487 ± 567	1544 ± 605	1542 ± 548	
**PWV,** m/sec	7.9 ± 2.1	8.7 ± 2.6	8.5 ± 2.3	8.7 ± 2.2	

BMI: body mass index; AIx: augmentation index; SBPao: aortic (central) systolic blood pressure; SBPbr: brachial systolic blood pressure; DBPbr: brachial diastolic blood pressure. HR: heart rate; SV: stroke volume; SVR: systemic vascular resistance; PWV: pulse wave velocity. Values are presented as mean ± standard deviation.

In linear regression analysis, age and gender explained 46% of the variation in AIx in the total study sample. Adding conventional CVD risk factors increased this to 54%. Next to age and gender, addition of body height, heart rate, SVR, SV and PWV explained 72% of the variations in AIx. Within each ethnic group, the contribution of the different physiological determinants to the variation in AIx was comparable (S1 Table). Table 2 shows differences in estimates of AIx among ethnic groups with correction for various covariates. Age and gender adjusted AIx was 8.2±0.4% higher in South-Asian Surinamese, 3.4±0.4% higher in African Surinamese and 6.3±0.4% higher in Ghanaians as compared with Dutch, all p<0.001. Additional correction for cardiovascular risk factors only slightly reduced the difference in AIx (model 2), whereas correction for physiological factors next to age and gender significantly attenuated ethnic differences in AIx (model 3). Correction for body height attenuated age and gender corrected ethnic differences in AIx the most to 4.4±0.4% for South-Asian Surinamese, 1.3±0.4% for African Surinamese, and 3.0±0.3% for Ghanaians, while PWV corrected differences in AIx to 6.1±0.3% for South-Asian Surinamese, 2.5±0.3% for African-Surinamese and 4.4±0.3% for Ghanaians as compared to Dutch. Correction for SV and SVR marginally attenuated ethnic differences in AIx, while correction for heart rate increased the differences in AIx between ethnic groups (data not shown). Additional correction for the use of antihypertensive therapy in both models 1 and 2 had a minor effect (<0.1%) on ethnic differences in AIx (data not shown).

**Table 2:** Difference in augmentation index (AIx) between ethnic groups.

		Difference ± SE	P-value
**Model 1**	*Dutch*	*(reference)*	
Age, Gender	South-Asian Surinamese	8.2 ± 0.4	p<0.001
	African Surinamese	3.4 ± 0.5	p<0.001
	Ghanaians	6.3 ± 0.4	p<0.001
**Model 2**	*Dutch*	*(reference)*	
Age, Gender, SBPbr, BMI, TC, smoking, DM	South-Asian Surinamese	7.9 ± 0.4	p<0.001
	African Surinamese	2.9 ± 0.3	p<0.001
	Ghanaians	5.5 ± 0.4	p<0.001
**Model 3**	*Dutch*	*(reference)*	
Age, Gender, Height, PWV, HR, SV, SVR	South-Asian Surinamese	3.9 ± 0.4	p<0.001
	African Surinamese	2.1 ± 0.3	p<0.001
	Ghanaians	3.0 ± 0.3	p<0.001

SBPbr: (seated, brachial) systolic blood pressure; BMI: Body Mass Index; TC: total cholesterol; DM: diabetes mellitus; PWV: pulse wave velocity; HR: heart rate; SV: stroke volume; SVR: systemic vascular resistance.

Irrespective of ethnicity, females (36±17%) had significantly higher AIx than males (26±16%), p<0.001. Correction for additional covariates (as in included in model 2 and 3) next to age revealed that height had the largest impact on gender differences in AIx. With correction for age and height the estimated gender-difference AIx was reduced by approximately 50%, to 4.9±0.3%, p<0.001.

Table 3 shows differences in AIx stratified by age category. In the young, AIx was significantly lower in Dutch (11.8±8.5%), compared to South-Asian Surinamese (14.5±8.7%), African Surinamese (14.2±10.3%) and Ghanaians (14.3±10.5%), all p<0.001. Stepwise, forward linear regression revealed height as the most important determinant of differences in AIx. After correction for height differences in AIx were all <1% in participants aged ≤30 years of age and no longer significant. In the elderly (age ≥60 years) AIx was lower in Dutch (37.9±14.9%) compared to South-Asian Surinamese (45.7±13.3%, p<0.001), African Surinamese (39.9±14.9%, p = 0.001) and Ghanaians (39.4±14.7%, p = 0.027). In South-Asian Surinamese, differences in height remained the most important contributor to differences in AIx, whereas differences in PWV were the most important determinant of AIx in elderly African Surinamese and Ghanaians.

**Table 3:** Ethnic difference in AIx in younger (age ≤ 30 years) and older (age ≥60 years) individuals.

		Difference ± SE	P-value
**Age ≤ 30 years**			
**Crude**	*Dutch*	*(reference)*	
	South-Asian Surinamese	2.7 ± 0.7	p<0.001
	African Surinamese	2.4 ± 0.7	p<0.001
	Ghanaians	2.6 ± 0.7	p = 0.001
Correction for Height:	*Dutch*	*(reference)*	
	South-Asian Surinamese	0.6 ± 0.6	p = 0.38
	African Surinamese	0.1 ± 0.6	p = 0.82
	Ghanaians	0.7 ± 0.7	p = 0.31
Correction for PWV:	*Dutch*	*(reference)*	
	South-Asian Surinamese	2.9 ± 0.6	p<0.001
	African Surinamese	2.4 ± 0.7	p<0.001
	Ghanaians	2.4 ± 0.7	p = 0.001
**Age ≥ 60 years**			
**Crude**	*Dutch*	*(reference)*	
	South-Asian Surinamese	7.7 ± 0.7	p<0.001
	African Surinamese	2.0 ± 0.6	p = 0.001
	Ghanaians	1.5 ± 0.7	p = 0.027
Correction for Height	*Dutch*	*(reference)*	
	South-Asian Surinamese	0.4 ± 0.7	p = 0.54
	African Surinamese	2.5 ± 0.6	p<0.001
	Ghanaians	4.5 ± 0.7	p<0.001
Correction for PWV	Dutch	*(reference)*	
	South-Asian Surinamese	4.1 ± 0.6	p<0.001
	African Surinamese	1.2 ± 0.5	p = 0.021
	Ghanaians	0.7 ± 0.6	p = 0.219

PWV: pulse wave velocity.

## Discussion

In the present study we show that there are substantial differences in AIx and central systolic BP between subjects from various ethnic backgrounds. In addition, we demonstrate for the first time that the differences in AIx and central BP between ethnic groups are largely independent of differences in traditional cardiovascular risk factors and are predominantly influenced by physiological determinants, particularly body height.

Our data are in agreement with previous studies that found higher AIx and central BP in subjects from African and South-Asian descent compared to people of Western-European descent.[11–14] Interestingly, ethnic differences in central systolic BP were entirely accounted for by differences in peripheral BP for African subjects, and by differences in AIx for South-Asian subjects. This implies that in particular in South-Asian subjects peripheral BP readings may underestimate the actual BP burden imposed to target organs because of increased wave reflection.

Body height and arterial stiffness explained most of the ethnic differences in AIx, while heart rate, which is inversely related to AIx,[18] increased ethnic differences in AIx because of the lower heart rate in Dutch. In line with our findings, Chirinos et al.[11] recently demonstrated that ethnic differences in AIx remained present after correction for a limited number of physiological determinants known to be associated with AIx, including height. By contrast we also performed concomitant assessment of the known hemodynamic determinants of AIx, (including PWV HR, SVR and SV) thereby enabling comparison of their relative contribution and found body height and large artery stiffness to be most important determinants. Body height and large artery stiffness affect arterial wave reflection by altering the timing between forward and backward travelling pressure waves.[16,20,33–35] Increased arterial stiffness causes faster propagation of pressure waves, while shorter stature may reduce both the travel path of the reflected pressure wave and travel time resulting from a decrease in aortic diameter.[36,37] In both instances the resultant is an earlier return of reflected pressure waves in the proximal aorta, leading to increased wave reflection and central BP augmentation. Although adult height has been shown to independently predict coronary artery disease and stroke,[38,39] the mechanisms leading to a higher risk of CVD are still undetermined. An increase in wave reflection and central BP could be one of the potential mechanisms linking body height to CVD risk.

In the present study, we demonstrate a divergent pattern of age-dependent rise in AIx among different ethnic groups. In young subjects, ethnic differences in AIx were principally driven by differences in body height, while PWV was not a predictor of ethnic differences in wave reflection. In subjects aged ≥60 differences in body height remained the strongest predictor followed by PWV. Because increased vascular stiffness was the driving force behind the age-dependent increase in AIx, ethnic differences in AIx that emerge with age most likely result from disparities in vascular ageing. By contrast, younger subjects may not have developed significant vascular remodelling as evidenced by the much smaller absolute difference in AIx. This suggests that disparate gene-environment interactions relevant to arterial stiffening, may be responsible. In addition, potential age dependent interactions between determinants of wave reflection could be different for subjects of different ethnic backgrounds thereby affecting disparities in AIx by age for various ethnic subgroups. Although we studied subjects from the same geographical area, migration history may have contributed to selection of participants. Differences in socio-economic, geographic and environmental factors have been shown to influence central BP and AIx.[40,41] In addition, there was a relative underrepresentation of elderly subjects of Ghanaian descent, which may have contributed to the apparent decrease in differences in AIx and central BP at older age. Otherwise, there was a remarkable concordance in the differences and determinants of wave reflection and central BP between African-Surinamese and Ghanaians. As both populations share a common West-African ancestry, this may imply a similar contribution of gene-environment interactions.

### Strengths and Limitations

The strengths of our study are the large scale, the fact that subjects were studied under identical geographical circumstances, and the concomitant investigation of various potential determinants of wave reflection. Potential limitations of the study are its cross sectional nature and the fact that differences in ethnicity and migration history may not be generalizable to other migrant populations. Non-invasive techniques were used to assess central hemodynamics and wave reflection. These techniques have been validated mostly in populations of West-European origin. Because the physiological determinants of the outcomes of interest were homogenous across different ethnic groups, we assume that the physiological principles are also generalizable to other ethnic groups. Arterial stiffness measured with the Arteriograph system shows comparable values as obtained by invasive measurements and by MRI.[42] However, in contrast to AIx, assessment of arterial stiffness using a one-point estimate has moderate correlation with the foot-to-foot method rendering extrapolation of absolute values difficult.[24] For the present analyses we used relative differences to assess the contribution of PWV to changes in AIx, although differences in body height remained the strongest predictor of ethnic differences in AIx, also in the higher age categories.

### Perspectives

Our study shows that AIx and central systolic BP are substantially increased in subjects from African and South-Asian origin compared to persons of West-European origin. Peripheral BP was the most important determinant of differences in central BP in African Surinamese and Ghanaians, whereas differences in AIx was the most important determinant of differences in central BP in South-Asian Surinamese. Body height contributed most to the ethnic differences in AIx. In contrast, conventional cardiovascular risk factors had a small contribution to ethnic differences in wave reflection. Because of the contribution of height to ethnic differences in AIx, our findings suggest that body height may be an important risk factor to explain part of the underestimation of cardiovascular risk observed in populations of African and South-Asian descent.

## Supporting Information

S1 Table Associations of physiological determinants with AIx for the total study sample and stratified by ethnicity. *β: indicates standardized regression coefficients.
(DOCX)

## Abbreviations

AIx: augmentation index
BP: blood pressure
CO: cardiac output
CVD: cardiovascular disease
HELIUS: healthy life in an urban setting
HR: heart rate
MAP: mean arterial pressure
PWV: pulse wave velocity
SV: stroke volume
SVR: systemic vascular resistance
