# Correction: Ethnic Differences in Arterial Wave Reflection Are Mostly Explained by Differences in Body Height—Cross-Sectional Analysis of the HELIUS Study

**DOI:** 10.1371/journal.pone.0168620

**Published:** 2016-12-15

**Authors:** Daan W. Eeftinck Schattenkerk, Jacqueline van Gorp, Marieke B. Snijder, Aeilko H. Zwinderman, Charles O. Agyemang, Ron J. G. Peters, Bert-Jan H. van den Born

In the Author Contributions section, Bert-Jan H. van den Born (BJHvdB) should be listed as one of the persons who conceived and designed the experiments.
