# Supplementary material for: Ethnic Differences in Arterial Wave Reflection Are Mostly Explained by Differences in Body Height - Cross-Sectional Analysis of the HELIUS Study
Source: PLoS One. 2016 Jul 29;11(7):e0160243. doi: 10.1371/journal.pone.0160243 (PMC4966932; doi:10.1371/journal.pone.0160243)
Supplement: S1 Table — *β: indicates standardized regression coefficients. (DOCX) [file pone.0160243.s001.docx]

**Online Supplement:**

|  | **Total study sample** | | **Dutch** | | **South-Asian Surinamese** | | **African Surinamese** | | **Ghanaians** | |
| --- | --- | --- | --- | --- | --- | --- | --- | --- | --- | --- |
| **Model fit** | R² **= 0.716** | | R² **= 0.747** | | R² **= 0.763** | | R² **= 0.678** | | R² **= 0.682** | |
|  | **β*** | **Sig.** | **β*** | **Sig.** | **β*** | **Sig.** | **β*** | **Sig.** | **β*** | **Sig.** |
| **Age**, years | 0.312 | <0.001 | 0.413 | <0.001 | 0.334 | <0.001 | 0.289 | <0.001 | 0.280 | <0.001 |
| **Female,** gender | 0.072 | <0.001 | 0.135 | <0.001 | 0.049 | 0.01 | 0.098 | <0.001 | 0.180 | <0.001 |
| **Height**, cm | -0.276 | <0.001 | -0.228 | <0.001 | -0.219 | <0.001 | -0.182 | <0.001 | -0.172 | <0.001 |
| **PWV**, m/sec | 0.441 | <0.001 | 0.434 | <0.001 | 0.426 | <0.001 | 0.438 | <0.001 | 0.423 | <0.001 |
| **HR**, beats/min | -0.297 | <0.001 | -0.275 | <0.001 | -0.258 | <0.001 | -0.345 | <0.001 | -0.318 | <0.001 |
| **SVR**, dynes/sec/cm^5^ | 0.134 | <0.001 | 0.091 | <0.001 | 0.161 | <0.001 | 0.134 | <0.001 | 0.229 | <0.001 |
| **SV**, ml | 0.031 | 0.001 | 0.035 | 0.042 | 0.064 | 0.005 | -0.010 | 0.55 | 0.086 | <0.001 |

PWV: pulse wave velocity ; HR: heart rate ; SVR: systemic vascular resistance ; SV: stroke volume.
